# Building capacity for collaborative research on opioid and other substance use disorders through the Clinical and Translational Science Award Program

**DOI:** 10.1017/cts.2019.441

**Published:** 2019-11-25

**Authors:** Linda B. Cottler, Alan I. Green, Harold Alan Pincus, Scott McIntosh, Jennifer L. Humensky, Kathleen Brady

**Affiliations:** 1 Department of Epidemiology, Colleges of Public Health and Health Professions, and Medicine, University of Florida, Gainesville, FL, USA; 2 Geisel School of Medicine, Dartmouth College, Hanover, NH, USA; 3 Irving Institute for Clinical and Translational Research and Department of Psychiatry, Columbia University, and New York State Psychiatric Institute, New York, NY, USA; 4 Center for Leading Innovation and Collaboration, University of Rochester, Rochester, NY, USA; 5 Department of Public Health Sciences, University of Rochester Medical Center, Rochester, NY, USA; 6 Medical University of South Carolina, Charleston, SC, USA

**Keywords:** Opioid use disorder, opioid use, CTSA Program, synergy, opioid crisis, NIDA, translational science

## Abstract

The opioid crisis in the USA requires immediate action through clinical and translational research. Already built network infrastructure through funding by the National Institute on Drug Abuse (NIDA) and National Center for Advancing Translational Sciences (NCATS) provides a major advantage to implement opioid-focused research which together could address this crisis. NIDA supports training grants and clinical trial networks; NCATS funds the Clinical and Translational Science Award (CTSA) Program with over 50 NCATS academic research hubs for regional clinical and translational research. Together, there is unique capacity for clinical research, bioinformatics, data science, community engagement, regulatory science, institutional partnerships, training and career development, and other key translational elements. The CTSA hubs provide unprecedented and timely response to local, regional, and national health crises to address research gaps [Clinical and Translational Science Awards Program, Center for Leading Innovation and Collaboration, *Synergy paper request for applications*]. This paper describes opportunities for collaborative opioid research at CTSA hubs and NIDA–NCATS opportunities that build capacity for best practices as this crisis evolves. Results of a Landscape Survey (among 63 hubs) are provided with descriptions of best practices and ideas for collaborations, with research conducted by hubs also involved in premier NIDA initiatives. Such collaborations could provide a rapid response to the opioid epidemic while advancing science in multiple disciplinary areas.

## Introduction

Collaborative and institutional infrastructure grants funded by the National Institutes of Health (NIH) offer unprecedented opportunities to address multiple issues affecting population health locally, regionally, and nationally. The opioid crisis is one major health issue significantly affecting the US population that could be urgently addressed utilizing resources and infrastructure of a national network. To address a crisis of this magnitude, a holistic approach is needed that covers the entire spectrum of clinical and translational research, from basic science and animal models to community engagement science [1]. Programs funded by the NIH National Center for Advancing Translational Sciences (NCATS) and the National Institute on Drug Abuse (NIDA) could be efficiently leveraged to speedily address the opioid crisis.

### The Opioid Crisis

NIDA is the NIH institute that funds the majority of research on substance use disorders and opioids in particular. The opioid epidemic has been responsible for an economic cost of at least $630Bf from 2015 to 2018. One-third of the cost is attributable to healthcare, 40% is attributable to early mortality, and 6% is from the criminal justice system with another 6% from child and family assistance. Finally, 15% is attributable to lost productivity [2–6].

The defining features of the opioid epidemic have changed over time with potent non-prescription illicitly produced fentanyl the cause of many opioid-related ER presentations and mortality because it is less expensive to produce than heroin [7–9]. In 2016, there were 42,249 deaths (115 per day) from opioids specifically and 63,632 deaths (174 deaths per day) from drug poisonings—a 21.4% increase from 2015, which is higher than deaths attributable to traffic accidents [10–12]. Two-thirds of these deaths were attributable to opioids [13]. With changing demographics, and a rising incidence of neonatal abstinence syndrome due to use of opioids during pregnancy [14], investigators understand the need to address not only opioid use disorder (OUD) but also substance use disorder (SUD) attributable to cannabis, stimulants (such as cocaine and meth) as well as vaping.

The healthcare system has been impacted from primary care, emergency medicine, and obstetrical physicians to pain medicine specialists [15, 16]. Back pain and arthritis are common conditions and are considered an epidemic with more than 100M people in the USA reporting recent or ongoing pain. In fact, pain conditions comprise four of the top six causes of disability in the USA, with back pain the leading cause [17, 18]. With societal costs of chronic pain in the USA over $600B, pain and opioid use are significant public health crises. Because pain is the primary reason for opioid use, which is necessary (though not sufficient) for OUD, it is paramount that attention is urgently paid to pain and access to opioids. Advancing knowledge in these arenas could be initiated through an integrated transdisciplinary approach spanning the bench to the community. One initiative that incorporates such a transdisciplinary approach is the NIH/NCATS Clinical and Translational Science Award (CTSA) Program.

### The CTSA Program

Clinical and translational research encompasses a broad continuum of research, moving scientific discoveries to clinical innovations that prevent or treat human disease [19]. Funded through NCATS, the CTSA Program was created in 2006 to facilitate and accelerate the translation of discoveries into new therapies to improve patients’ health and well-being. Through a national network of academic health and research centers called “hubs,” the program supports the development of innovative treatments that safely reach patients and communities faster. Each hub supports a local and regional clinical and translational research infrastructure, encompassing clinical research, bioinformatics, data science, community engagement, regulatory science, training and career development, institutional partnerships, and other key elements of the translational science spectrum. Deeply rooted in the CTSA Program mission is the education and training of the next generation of clinical and translational scientists using innovative techniques and tools with an emphasis on collaboration and team science, specifically accomplished through the CTSA KL2 and TL1 programs, and other training activities within the network.

As a national network connecting people to meaningful research, with a 21st century infrastructure, the CTSA Program hubs serve their regions to initiate a timely response to local, regional, and national health crises. The combined capacity of the CTSA Program hubs—infrastructure and expertise—is proving to be ideal for deploying and implementing innovative therapies and tools to address the opioid crisis. Thus, the CTSA Program has produced initial work in pain management and opioid research, including community engagement; however, as the response to this crisis continues, efforts must maximize limited resources and develop a synchronized approach that considers existing strengths and expertise [e.g., medication-assisted treatment (MAT), data science, recruitment and retention, EHR technologies, community, pediatrics] while maintaining each hub’s local/regional context [20–22]. In this context, the CTSA Program facilitates the development of synergistic collaborations to efficiently test and implement meaningful translational research projects to solve specific problems across the translational spectrum. Because hubs are disease “agnostic,” their networks include all areas of medicine, surgery, nursing, pharmacy, communication science, biomedical informatics, psychology, anthropology, epidemiology, library science, and others that reside in university-based academic medical centers. As such, if collaborative resources were marshalled, the impact could be multiplicative and profound.

### The Effort to Collaborate

This paper describes the landscape of opioid research with CTSA Program hubs with the goal of creating opportunities to build capacity to promote best practices and innovative collaborative research related to opioids and eventually other substances or crises requiring a synergistic approach. This “synergy” paper offers ideas for collaborative growth and science as this crisis progresses and different trends emerge. The hope is that the NIH, foundations, industry, and other entities will facilitate research and the development of new therapeutic options for opioid (and other disorders) research to improve population health.

## Methods

To move the needle forward, NIDA and NCATS leadership and hub scientists outlined gaps and initiatives that hubs could strengthen together. These initiatives were planned in three phases.

### Phase 1: Conference Meetings

NIDA leadership spearheaded three conference meetings in summer 2017 that focused on medication development for OUD and overdose (OD), the development of safe and non-addictive medications for pain relief, and neurobiological mechanisms of pain. In June 2018, the Center for Leading Innovation and Collaboration (CLIC), the CTSA Program coordinating center at University of Rochester, hosted a consortium-wide “Un-Meeting” on the opioid crisis with scientists and external stakeholders across the translational spectrum [23]. The event featured brief presentations from experts with a group discussion of issues such as non-pharmacological pain management, implementation science, high-risk youth and other populations, telemedicine, community engagement, and health policy.

### Phase 2: Virtual Meetings and Working Group

In Phase 2, the CTSA Program Steering Committee focused on the crisis in July 2017. Subsequently, NCATS and NIDA held a 2 hour virtual meeting August 2017 with representatives from the CTSA Program hubs with expertise in substance abuse and/or pain. NIH Directors Drs. Volkow (NIDA) and Austin (NCATS) presented data and facilitated a discussion on potential collaborations, which was followed by the formation of an opioid interest workgroup October 2017 with representatives from CLIC, NCATS, NIDA, and eight CTSA Program hubs; they planned a Landscape Survey of activities and capacity to address the opioid epidemic.

### Phase 3: Landscape Survey of the Hubs to Fill Research Gaps and Collaborate

To build and strengthen research capacity between hubs, the CTSA Program Steering Committee Opioid Workgroup, facilitated by CLIC, developed the HIPAA compliant online Landscape Survey using an iterative pretesting process involving experts and other stakeholders.

The goal of the Survey was to generate a broad landscape analysis of current opioid-related programs representing either best practices or innovative programs underway across the CTSA Program hubs, individually and with diverse partners. Principal investigators (PIs) at each hub asked the person most knowledgeable at their site to complete the Survey by April 30, 2018.

Procedures were established to systematically and iteratively code the qualitative findings from the open-ended text-based survey item data. All quotes and passages provided by respondents were structurally organized by the study team [24]. Two coders were initially assigned a domain and reviewed the text. Initial codes were developed that defined, labeled, and promoted inclusivity of the text-based data. After establishing a broad framework for data analysis, axial coding (a structured process to associate self-reported constructs) consolidated text [25]. The Workgroup met virtually to debrief and compare emerging data patterns in order to solidify codes (themes). Text-based data were sorted and reviewed by the Workgroup.

After coding, review, and regular peer debriefing, themes that emerged were further organized within broader domains when applicable and compared among and between domain areas during additional meetings [26]. Finally, two co-authors, LBC and SM, consolidated the domains and text over six sessions to increase confidence in the fidelity of the qualitative methods and the trustworthiness of the resultant themes [27–29].

## Results

### Identification of Areas of Strength

Several gap areas were outlined during the study phases (Table 1). These included understanding the neurobiology of pain management, best practices in clinical management of pain, developing new and innovative medications and technologies for better addiction treatment, improving access to efficacy-based addiction treatment, and reducing and reversing ODs. One role of the CTSA Program was to understand the capacity for efforts to work with systems such as law enforcement, criminal justice, surgical and pain specialists, emergency departments, neonatal units, and the community at large.

**Table 1.** Research priority areas of National Institute on Drug Abuse (NIDA) and National Center for Advancing Translational Science (NCATS)

Research domain	Priority areas
*Pain management* (safe and more effective strategies)	Non-opioid analgesics: cannabinoids, anti-inflammatory medications, ion channel blockers
	Targeted opioid analgesics
	Biologics: antibodies that bind to pain producing cytokines
	Non-pharmacologic treatments: Neural stimulation; surgical intervention, meditation, etc.
*Opioid addiction treatment* (new and innovative medications and technologies)	Mapping differences in the brain
	Medication-assisted treatment (MAT)
	Emergency department initiated buprenorphine
	Naltrexone treatment for criminal justice populations
	Fentanyl vaccine
*Overdose reversal* (interventions to reduce mortality and linkage to treatment)	More widespread naloxone distribution
	Stronger, longer acting formulations for more potent opioids
	Devices to stop respiratory depression
	Overdose detection and alert technologies
	Linkage to treatment after over dose (OD)

### Hub Capacity

Results of the Landscape Survey from the Phase 3 consolidation uncovered numerous ideas to fill the knowledge gap, identify models to disseminate, and provide opportunities for collaborations. The Survey casts a wide net in that the 47 respondents were inclusive of PIs of the CTSA Program hubs (37%; *n* = 17), Administrators at the hubs (38%; *n* = 18), and Managers, Evaluation Specialists, Researchers, and Program Directors (25%; *n* = 12). After data cleaning and direct confirmation of sites regarding specific information, a final dataset reflected responses from 45 of 63 hubs active during FY17, including those both currently funded and in a no cost extension phase, representing a 70.3% response rate [30]. Those who filled out the Survey did have different areas of capacity, but were deemed to be those who could best communicate ongoing efforts at their hub. Salient items per topic, that is, the items that were reported by the responder, along with the number of hubs reporting them, are shown in Table 2. Although instructions in the survey requested data about activities at the hub, some identified training opportunities, research, collaborations, or best practices from activities at their institutions, whether related to their CTSA or not.

**Table 2.** Capacity by domain, activity type, general approach, and number of Clinical and Translational Science (CTSA) program hubs mentioning that domain among the 45 hubs that responded

Capacity	Activity/topic	General approach examples	Number of hubs mentioning this
Pre-clinical	Chronic pain	Noninvasive neuromodulation techniques, Mu agonist delta antagonist opioids, mechanism of nociception induced by innocuous cold in trigeminal system, pain induction, G protein-coupled receptors and transient receptor potential ion channels, self-reported pain, pharmacological and non-pharmacological treatments, herbal remedies	8
	Biomarkers/Imaging	Psychophysical and brain imaging techniques, functional magnetic resonance imaging (fMRI), magnetic resonance (PET/MR) imaging, blood oxygen level dependent (BOLD) fMRI, arterial spin labeling (ASL), integrated positron emission, imaging of novel compounds	3
	Novel approaches	Brain plasticity to mediate opioid seeking behavior in conditions of pain, microassays for use in infants and newborns, transcranial direct current stimulation (tDCS), and transcranial magnetic stimulation (TMS) in pain	3
	Genetics	Genomics, genetics of pain and opioid analgesia, gene therapy platforms	2
	Animal models	Brain–computer interface in animal models, animal models of pain in burns	2
Clinical	Recruitment and retention/screening	Social media, community engagement, patient research partnership; rural research networks, electronic data warehouse (EDW), wearable technologies, health claims data, telehealth, retention efforts, dashboards to facilitate partnership with patients, social network analysis, electronic medical record (EMR) based screening/identification	13
	Pain management	Emergency department (ED) initiated opioid and non-opioid prescribing patterns, perioperative pain management, cognitive behavioral therapy (CBT), multi-disciplinary pain rehabilitation, rural telehealth, opioid prescribing patterns; alternatives to opioids (ALTO): nutritional and herbal supplements, meditation, cognitive behavioral therapy (CBT)	11
	Medication-assisted treatment (MAT)	Outcomes of collaborative care models in primary care settings, suboxone clinics, methadone maintenance treatment, low dose naltrexone, buprenorphine/naloxone, specialty care	5
	Perinatal/Obstetrics	Perinatal and mother’s treatment programs, pregnant women with opioid use disorder (OUD), neonatal abstinence syndrome (NAS), rural telehealth	3
	HIV	Persons living with human immunodeficiency virus (PLWHIV), outcomes among human immunodeficiency virus (HIV) infected opioid-dependent patients receiving buprenorphine/naloxone	1
Community engagement	Community networking	Community Advisory Boards (CABs)	Nearly all sites
		Community academic partnerships	3
		Social media/Machine learning	3
		Traumatic brain injury (TBI) network	1
		Health affairs blog	1
	Local/Regional/National town hall meetings	Our community our health (national)	1 + 22 CTSI sites
		Conference on opioids	4, CDC, NIH
		Science cafes	1
		Community conversations about the opioid epidemic	1
	Sharing of data with state, local, and government leaders	With Governor, President’s Opioid Task Force, Mayor, Department of Health (DOH), Surgeon General, Opioid Task Force, Medical Examiner, State Opioid Response, harm reduction	Nearly all sites
		Hot-spotting geospatial techniques to identify areas of risk	5
	Community engagement programs	Community linkages	2
		Community engagement program with community health workers	2
	Harm reduction community outreach program	Needle exchange	2
		Deterra deactivation pouch distribution	2
		Narcan distribution	2
	Needs assessments	“All of Us”	3
		Stakeholders, community members	2
		Opioid risk tool – ED	2
Data science	Enterprise data warehouse	Data warehouse, i2b2 query to identify patients with opioid prescriptions	Nearly all sites
	Health claims databases	Overdose data; analysis of electronic record data; prescribing studies of insurer databases	Nearly all sites
	Prescription drug monitoring program (PDMP)	Statewide effort	Nearly all sites
	Electronic health records data	Opioid prescription data, high-risk patients, natural language processing (NLP), epidemiology and rural service accessibility, telemedicine, registries, phenotypes	11
	Predictive modeling	Network-based models, machine learning, public–private access database	11
	Cloud-based technologies	Clinical data to address OUD	1
Workforce development	Clinical training	Face to face, community-based learning, eHealth platform, virtual reality, collaboration with outside entities [e.g., Project Extension for Community Healthcare Outcomes (ECHO)]. Training on: MAT, prescribing, quality improvement (QI) projects, for clinicians, clinical staff, residents, dentists, nurses, pharmacists, pharmacy residents, community agencies, law enforcement, and students (dental, pharmacy, and public health	27
	Clinical training and research training	Workshops, seminars, grand rounds, online training, decision support tools, web-based self-management and coaching, patient-centered educational programs to reduce the development of chronic pain and OUD	12
	Research training	Fellowship/certification programs; opioid use trends; student design competition	9

Five domains emerged and included: (1) *pre-clinical programs* focused on OUD and/or OD including research and development of chronic pain treatments; (2) *clinical research* on the development of innovative approaches and best practices to identify, recruit, and retain populations with OUD more effectively. This also included clinical trial networks that could be utilized for specific studies with surgical, adolescent, pain management, primary care, or neonatal populations, among others; (3) *community engagement* efforts that highlighted outreach, active partnerships with Departments of Health, Emergency Departments, community members, and others; (4) *computational science and data informatics*, distilled to “data science” which included effective approaches and epidemiological tools to affect the use of clinical data to address OUD and/or OD; and (5) training approaches to assess and address high risk use of opioids within the medical setting, under the umbrella of *workforce development and implementation science*.

In the pre-clinical domain, there were multiple mentions of research projects/programs related to chronic pain, from proteins to nociceptive mechanisms (eight hubs). The next most salient theme was related to biomarkers/imaging studies with three east coast hubs mentioning work in this area, followed by basic science studies relative to genetics (two hubs); other approaches were related to brain plasticity and brain–computer interfaces (two hubs each).

In the *clinical research* area, a number of key programs were enumerated. Most salient was research infrastructure related to recruitment, retention, and screening methods especially for high-risk populations. This effort included cohort discovery through social media, electronic health records, wearable technologies, and community outreach (mentioned by 13 hubs). This was followed by pain management research (11 hubs), MAT such as buprenorphine or methadone treatment (MAT-5 hubs), perinatal/obstetrics research (3 hubs), and HIV-related efforts (1 hub). The pain management programs ranged from research on nutritional and herbal remedies to alternatives to opioids.

*Community engagement* was a key focus of the hubs—especially reaching diverse populations. Nearly every site that responded mentioned their Community Advisory Board. Many other innovative efforts are underway, including Our Community, Our Health—a national town hall meeting series pertaining to issues that are a priority to the community—spearheaded by the University of Florida—involving 23 hubs to date. Along these same lines are the regional conferences on opioids and hub involvement in their state’s opioid response. Hubs are also conducting needs assessments, Community Engagement programs such as HealthStreet, Science Cafes, and harm reduction efforts, such as needle exchange and distribution of naloxone and medication deactivation pouches [31]. In this capacity, the CTSAs have transformed the landscape and contributed to the science of Community Engagement, elevating Community Engagement to “crown jewel” status. As expected, nearly all CTSA Program hubs mentioned at least one initiative in this area, including geospatial hotspot mapping.

The CTSA Program has also transformed the culture at most hubs to conduct highly innovative computational bioinformatics research in the area of *Data Science*. It has introduced the required need for data warehouses, honest brokers, querying tools for cohort identification, computable phenotypes, and predictive modeling. This area has involved the contributions of numerous and varied disciplines from computer science, bioinformaticians, mathematicians, statisticians, epidemiologists, and IT teams.

Finally, there is an extraordinary capacity for *workforce development* for translational research within the CTSA Program hubs. This key component provides training and continuing education on best practices to address public health issues. The information provided in this area was particularly difficult to codify because it varied so greatly. Training practices focused on clinical training, research training, or both. Clinical training included health professionals across a number of disciplines and specialties, including internal medicine physicians, psychiatrists and other prescribers, nurses, pharmacists, dentists, and public health professionals. It also included training for partners operating outside the university sector, including community-based agencies and law enforcement professionals. CTSA training activities covered topics such as general knowledge about the opioid crisis, treatment strategies, OD management/naloxone administration, MAT, pain management, and safe prescribing practices. Training activities encompassed a number of formats, including face-to-face, online (distance learning) formats, team science approaches, and collaborations with external entities, such as Project ECHO from the University of New Mexico, which provides tele-mentoring and training to community providers in the treatment of complex conditions [32]. Research training included fellowships and certification programs targeting medical students, graduate students, fellows and junior researchers. One particularly innovative approach included design competitions to enable students to design crisis solutions and share with policymakers. More general training methods included university events such as workshops, seminars, and grand rounds, as well as online trainings and web-based coaching. Other initiatives involved the development of decision support tools and patient education materials.

### Models of Care

As noted in Table 3, our respondents noted distinct models of care, focusing on a broad array of targeted individuals in many different clinical and non-clinical settings. Many CTSA Program hubs described interventions for OUD that involved linkage to care and dual disorder models, prescribing safety, communication strategies, naloxone distribution, case management, criminal justice, MAT, telehealth, collaborative care models, CHWs, nurse care models, statewide models, and others.

**Table 3.** Specific models of care, settings, and populations

*Models of care*		
Health disparities	Linkage to care models	Dual disorder
Prescribing safety	Communication strategies	Naloxone distribution
Case management	Criminal justice systems	Medication-assisted treatment (MAT)
Telehealth	Collaborative care models	Community health worker models
Statewide models	Nurse care managers	

*Populations*	*Settings*
First responders	Rural counties
College students	Primary care practices
Pregnant women	HIV clinics
Pain patients	College campuses
Underserved populations	Tribal settings
Populations that inject drugs	Psychiatric clinics
Veterans	Federally qualified health centers (FQHCs)
Faith-based communities	Drug Courts
Inmates/Drug court	Practice-based research networks (PBRNs)
Persons living with HIV/AIDS	Emergency departments (EDs)/Urgent care facilities
Persons with traumatic brain injury	Surgical clinics
Persons with history of domestic violence	Hospitals
Pre- and post-operative clinics	Construction sites
In-patients	Consensus meetings
Welders/Blue collar workers/Laborers	Town hall meetings
10–18 year olds	Pharmacies
Medical/Dental, nursing, pharmacy, public health students	Veterans affairs (VA)
Interns/Residents	
Law enforcement	
Medicare/Medicaid	

Data from the survey also highlighted that the CTSA Program hubs work *with and for* a number of important diverse populations, many of which are underrepresented in clinical and translational research. These are identified in Table 3, in no particular order. They include pediatric populations through older adults. Hubs are including first responders, college students, pregnant women, persons with pain, underserved populations, persons who use illicit drugs or who inject drugs, veterans, faith-based communities, inmates, persons in Drug Court, persons living with HIV/AIDS, with traumatic brain injury, persons in law enforcement, and many others.

### Training, Network, and CTSA Program Synergy

**Fig. 1.**
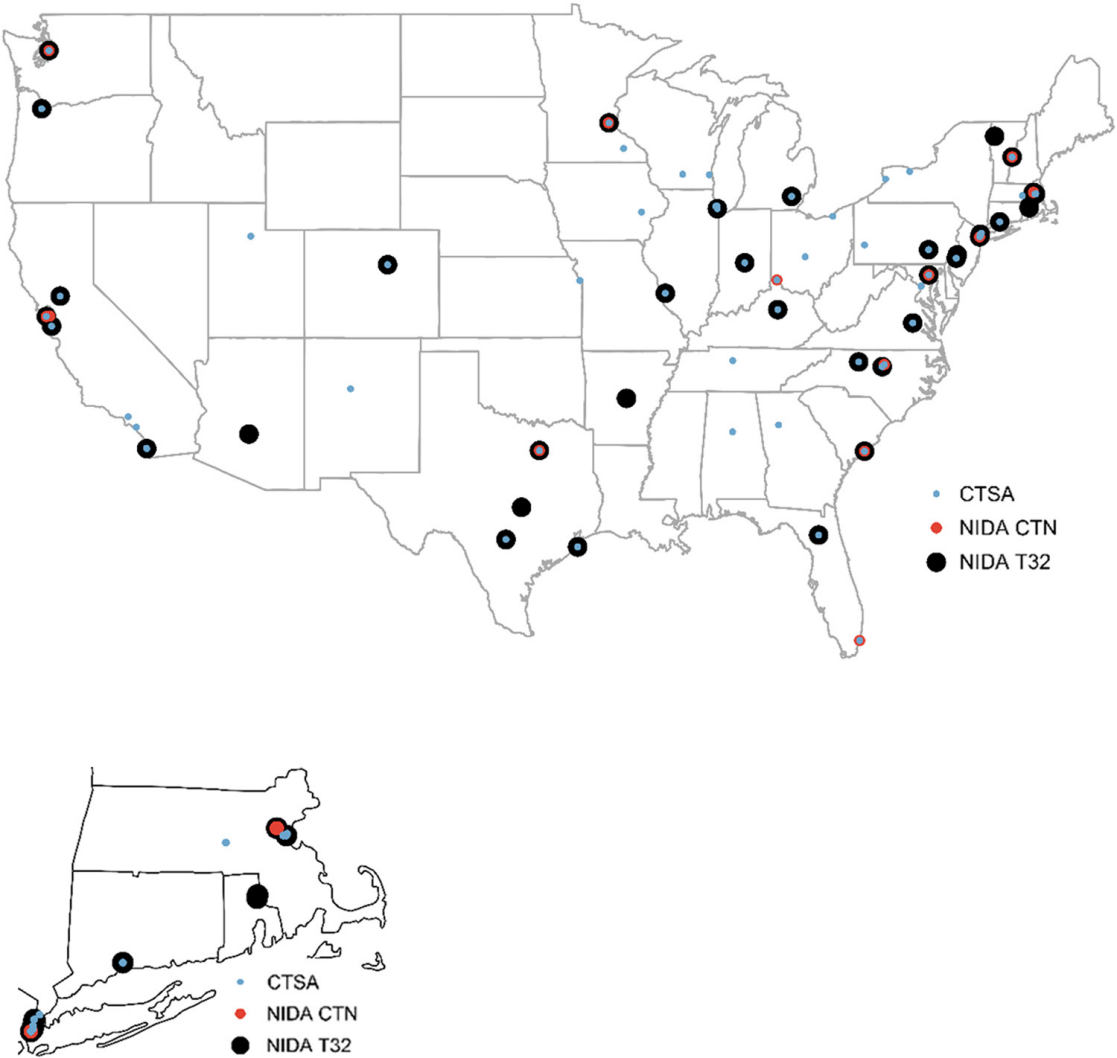
US Mainland Funded Locations by Clinical and Translation Science Awards (CTSA), National Institute on Drug Abuse (NIDA) T32s or Clinical Trials Network (CTN) funding in 2017–2018.

Lastly, to indicate the capacity of the CTSA Program to train the next generation of scientists, and to develop and promote translational science, we mapped the location of NIDA NRSA Training Grants (T32s) and NIDA Clinical Trial Networks (CTNs) in relation to the academic centers awarded a CTSA. Among the 62 CTSAs listed in the NIH Reporter (those in NCE and those awarded), 28 (45%) of the CTSA hubs had no CTN or T32; 24 (39%) had a T32 but not a CTN; 7 (11%) were the recipient of both a CTN and T32, and 3 (5%) of CTSA hubs had a CTN. As shown in Fig. 1, the CTSA Program hubs with both a T32 and CTN are depicted with a large black circle with two inner circles—one blue and one red [33]. Two of these seven hubs were on the west coast, one was in the Midwest, and four were on the east coast (with two in the northeast). The 28 hubs without a CTN or T32, depicted by a blue circle, tended to be isolated throughout the USA. CTSA hubs with a T32 (*n* = 24) but no CTN tended to be in the Midwest, the southeast, and northeast. The hubs with at least one other NIDA project (CTN/T32) (55%; *n* = 34) were considered to have maximal synergy for SUD research.

Additionally, there are 21 sites across the country, most of which are also aligned with CTSA Program hubs, with NIH funding for the “ABCD study”—the NIH funded Adolescent Brain and Cognitive Development longitudinal study of over 11,000 children enrolled at 9–10 years of age, who are being followed for 10 years [34]. These sites, with their Coordinating Center and Data Analysis and Informatics Center, have expertise in recruitment and retention, child and parent assessments, data coordinating expertise, and sharing of image and assessment data.

## Discussion

This first synergy paper from the NIH CTSA Program indicates a high degree of opportunity relevant to a serious scientific response to a national drug abuse crisis. NIDA and NCATS worked together to evaluate the gaps in the field, and the capacity at the CTSA hubs to field studies focused on opioids and eventually other drug trends.

Through three phases of initiatives that spanned conferences, working group calls, and a Landscape Survey, including an NIH Reporter analysis of built capacity through NIH awards, it was heartening to see that the CTSA Program has begun to change the landscape and reached out to improve the health of all through activities included in important domains: pre-clinical, clinical, community engaged, data science, and workforce development domains, with multiple themes in each. It must also be noted that many of these discussion points are the focus of conferences in which the researchers may or may not even know that the research is related to a CTSA Program hub. Of course, the Association for Clinical and Translational Science (ACTS) meetings is CTSA focused; however, the College on Problems of Drug Dependence hosts many scientists and numerous scientific contributions, in which an NCATS CTSA project might not be reported. If researchers acknowledged all funding sources during their conference presentations, it could foster collaborations across hubs and raise awareness of CTSA programs.

Additional discussion points relate to our metrics for success which we could adopt and monitor in an ongoing manner to track capacity building, close the gaps, and make headway toward reducing ODs, finding satisfactory alternatives to opioids, and treating OUD. Now that the capacity for research focused on many critical aspects of the OUD epidemic has been identified, it can be translated to focus on other SUDs.

Hubs could eventually evaluate their achievements in some of these areas: (1) hubs working together on both externally and internally funded initiatives, including cooperative agreements from NCATS as well as Un-Meetings on Rural Health or Regional Hub meetings; (2) the number of collaborative networks established and sustained over time; (3) the number of investigators, research coordinators, and others trained to conduct opioid research through the Translational Workforce Development programs; (4) specific models of care at each CTSA hub; (5) investigators trained in SUDs through K and T programs that have a NIDA T32 and how integrated the T32 is at each hub; (6) the mentoring mosaics that were established include an investigator from another hub; (7) increased pilot studies funded by hubs that are relevant to SUDs, in addition to opioids, and their risk factors; (8) the number of educational certificates launched that are relevant to OUD; (9) the number of grants that are submitted as well as funded that are pertinent to OUD and other SUDs; (10) community involvement regarding solutions related to OD; (11) the number of town hall meetings that involve discussions about substance use and its harm, and (12) an increase in the percentage of people with substance use or SUDs who are both recruited into and retained in health research. Metrics such as these as well as other morbidity and mortality statistics could be evaluated and tracked [35, 36]. Moreover, with time, surveillance could note how the institutional culture was transformed at each site regarding the points enumerated above, including how each of the initiatives helped to inform the other. For example, what community engaged efforts determined the focus of clinical trials or pilot grants.

Collaboration could also be facilitated through the Trial Innovation Network (TIN), established by NCATS in 2016. The TIN focuses on research to establish methods to improve the clinical trials process and enhance the network capacity of the CTSA Program. Three Trial Innovation Centers, one Recruitment Innovation Center, and the CTSA Program hubs comprise the TIN. The TIN offers resources to support investigators and their research teams, such as single IRB reviews, assistance setting up master clinical trial agreements, advice on protocol design, participant recruitment and retention plans, help finding additional CTSA clinical sites for studies, and serving as the data coordinating center for funded studies. The TIN multisite clinical research infrastructure can provide targeted support for studies that need to be implemented in a rapid and efficient manner, such as studies that address the opioid epidemic.

An important limitation must be noted. Some hubs may have identified only CTSA activity while others may have provided information about all activities at their home institution regardless of funding. This discrepancy reflects a conservative outlook as to the complete universe of capacity at each site. Benefit of the doubt should be given to all hubs—they should each be viewed as worthy partners in any collaborative approaches. In fact, now that the domains have been established, it is feasible for the Workgroup to repeat the survey in an informed context and with intentionality to plan for multisite studies.

This limitation notwithstanding, there were a number of strengths found through this landscape analysis, primarily the robust enumeration of the capacity available at each site. While sites may not have known until this time of all the initiatives available to them at each site, or even their own, they can now be fully aware of the initiatives to be brought to bear to address this topic locally, regionally, and nationally. In fact, the NIH Helping to End Addiction Long-term (HEAL) and its initiative offers additional, multiple mechanisms for CTSA Program hubs to consolidate efforts and collaborate.

## Conclusion/Next Steps

It is critical to marshal the efforts of the scientific community to deliver effective and sustainable solutions to this formidable public health challenge. Congress added $945M to the base appropriation of the NIH starting in fiscal year 2018 to invest in resources to support science that advances addiction and pain research. This new trans-NIH initiative—HEAL—is focused on identifying new treatments for addictions and non-addictive treatments for pain, better understanding of the mechanisms underlying pain and optimizing the use of effective treatments for addiction, including the development of new models of care [37]. The HEALing Communities mechanism was awarded to four sites—all of which are CTSA hubs and their capacity to address the issues with community input is based on the CTSA community focus that utilizes the principles of community engagement, developed by CTSA scientists, to advance the science of community engagement [38]. Finally, many of our CTSA hubs are involved in the National Academy of Medicine Action Collaborative on Countering the US Opioid Epidemic, which could provide networking opportunities in the areas we have highlighted in this synergy paper. A Phase 4 plan is the next step—one that is well-intentioned, one that is well-equipped, one that expands to other substances besides opioids, and one that utilizes the considerable expertise and talent of the NIH CTSA Program scientists with links to co-located NIDA T32 and CTN awards. A follow-up initiative should consider a venue such as the ACTS annual meeting where hub investigators get together to plan new collaborations.
